# Deregulation of microRNAs Let-7a and miR-21 mediate aberrant STAT3 signaling during human papillomavirus-induced cervical carcinogenesis: role of E6 oncoprotein

**DOI:** 10.1186/1471-2407-14-996

**Published:** 2014-12-23

**Authors:** Gauri Shishodia, Gaurav Verma, Yogesh Srivastava, Ravi Mehrotra, Bhudev Chandra Das, Alok Chandra Bharti

**Affiliations:** Division of Molecular Oncology, Institute of Cytology and Preventive Oncology, I-7, Sector-39, Noida, Uttar Pradesh 201301 India; Dr. BR. Ambedkar Centre for Biomedical Research, University of Delhi, Delhi, India

**Keywords:** HPV, Cervical cancer, microRNAs, STAT3, miR-21, Let-7a

## Abstract

**Background:**

Aberrantly expressed and constitutively active STAT3 signaling plays a pivotal role in initiation and progression of human papillomavirus-induced cervical carcinogenesis. However, the underlying mechanism(s) responsible for pleiotropic effects of STAT3 signaling is poorly understood. In view of emerging regulatory role of microRNAs, Let-7a and miR-21 that may interact with STAT3 signaling and/or its downstream effectors, present study was designed in HPV16-positive cervical cancer cells to assess the functional contribution of these miRs in STAT3 signaling in cervical cancer.

**Methods:**

Functional silencing of STAT3 signaling and HPV16 oncoprotein expression in SiHa cells was done by STAT3-specific and 16 E6 siRNAs. Pharmacological intervention of STAT3 was done using specific inhibitors like curcumin and stattic. Loss-of-function study of miR-21 using miR-21 inhibitor and gain-of-function study of let-7a was done using let-7a mimic in SiHa cells.

**Results:**

Functional silencing of STAT3 signaling in SiHa cells by STAT3-specific siRNA resulted in a dose-dependent decrease in cellular miR-21 level. Pharmacological intervention of STAT3 using specific inhibitors like curcumin and Stattic that abrogated STAT3 activation resulted in loss of cellular miR-21 pool. Contrary to this, specific targeting of miR-21 using miR-21 inhibitor resulted in an increased level of PTEN, a negative regulator of STAT3, and reduced active pSTAT3 level. Besides miR-21, restoration of cellular Let-7a using chemically synthesized Let-7a mimic reduced overall STAT3 level. Abrogation of HPV oncoprotein E6 by specific siRNA resulted in increased Let-7a but loss of miR-21 and a correspondingly reduced pSTAT3/STAT3 and elevated the level of cellular PTEN.

**Conclusions:**

Our results demonstrate existence of a functional loop involving Let-7a, STAT3 and miR-21 which were found potentially regulated by viral oncoprotein E6. Implications: miR-21 and Let-7a along with STAT3 may prove useful targets for pharmacological intervention for management of cervical cancer.

**Electronic supplementary material:**

The online version of this article (doi:10.1186/1471-2407-14-996) contains supplementary material, which is available to authorized users.

## Background

A high level of constitutively active STAT3 is a characteristic feature of many epithelial cell malignancies that include cervical cancer [1]. Aberrantly expressed and constitutively active STAT3 signaling plays a pivotal role in initiation and progression of cervical cancer and controls expression of viral oncogenes, E6 and E7 during cervical carcinogenesis [2, 3]. However, the underlying mechanism(s) responsible for pleiotropic effects of STAT3 signaling is poorly understood. Active STAT3 has multiple effects on cellular physiology and oncogenesis through transcriptional switching of several promoters of genes associated with malignant transformation [4]. Apart from a direct transcriptional control, recent studies suggest STAT3 may exert its oncogenic role through controlling the expression of microRNA [5]. Involvement of miRNA, particularly for fine tuning of transcriptional response, has been documented for almost all major cellular functions such as cell proliferation, cell differentiation, stress response, apoptosis and transcriptional regulation [6]. Accumulating evidence suggests potential involvement of a small subset of miRNAs in initiation and progression in a wide range of human cancers including cervical cancer [7–12]. miRNAs cooperatively function with transcription factors in the regulation of sets of target genes, allowing coordinated modulation of gene expression, both transcriptionally and post-transcriptionally. Our recent observation demonstrate a strong association of elevated miR-21 expression with active STAT3 and an inverse correlation with level of Let-7a in tumor tissues from cervical cancer lesions (unpublished data). These observations prompted us to investigate if an active Let-7a-STAT3-miR-21 functional signaling loop operates during cervical carcinogenesis.

Recent reports suggest that miR-21functions as an oncomiR in human cancers. Inhibition of miR-21 resulted in cell growth inhibition and caspase-dependent apoptosis in different types of cancer cells [13]. The gene encoding miR-21 is controlled by an upstream enhancer containing two STAT3 binding sites that are strictly conserved [14]. On the other hand, miR-21 targets PTEN gene through a binding site on the 3′UTR in hepatocellular carcinoma [15]. PTEN is a critical tumor suppressor gene that negatively regulates STAT3 activity [16]. Nevertheless, there is no evidence to support that miR-21 directly interact with STAT3 signaling. Moreover, the correlation, if any, of miR-21 expression with constitutively active STAT3 in cervical carcinogenesis is yet to be established. Apart from miR-21, another miRNA Let-7 was reported to interact with STAT3 signaling. STAT3 3′UTR possesses a strong putative Let-7a binding site [17]. Interestingly, Let-7a is frequently down-regulated in many human cancers including tumors of colon, lung, and breast [18, 19], and forced expression of Let-7 family members were found to suppress cancer cell growth, both *in vitro* and *in vivo* [20]. These studies suggested a potential tumor suppressive role of Let-7a. However, how Let-7a is involved in post-transcriptional regulation of STAT3 needs to be explored further.

With a particular reference to cervical carcinogenesis, which is caused by infection of high risk-HPVs through expression of their viral oncoproteins E6 and E7 [21], it was recently noted that STAT3 signaling plays a functional regulatory role [3] and get controlled by oncoprotein E6 [22]. In view of emerging regulatory role of microRNAs, Let-7a and mR-21 that may interact with STAT3 signaling and its downstream effectors, present study was designed in HPV16-positive cervical cancer cells to assess the functional contribution of these miRs in STAT3 signaling in cervical cancer. We also studied the effect of E6 silencing on miR-21 and Let-7a pools in cervical cancer cells. Results presented in this article demonstrate for the first time a relation between miR-21 and Let-7a in HPV E6-mediated active STAT3 signaling in cervical cancer cells.

## Methods

### Materials

STAT3 or HPV16 E6 siRNAs were procured from Santa Cruz Biotechnology (Santa Cruz, CA, USA) as pools containing 3–5 different target-specific 19-25 nt siRNA to non-overlapping sequences, along with scrambled siRNA which was used as control. RNAiMax transfection kit used to make transient siRNA transfection was procured from Invitrogen (Carlsbad, CA, USA). Commercially available STAT3 inhibitors, Stattic (STAT3 inhibitor 5; Calbiocam, USA) or herbal derivative curcumin or difurulylmethane (Sigma, St Louis, MO, USA) were dissolved in DMSO as stock (20 mM) and diluted in the medium immediately before use. miR-21 inhibitor and Let-7a mimic which are RNA oligonucleotides with novel secondary structure that are designed to inhibit and augment the function of endogenous miRNAs respectively were purchased from Dharmacon (Lafayette, CO, USA). miR-specific inhibitor and mimic were dissolved in nuclease-free water prior to use as per manufacturer’s instructions and were kept in aliquots at −20°C until use. Specific antibodies to STAT3 and enhanced chemiluminiscence (ECL) detection kit were purchased from Santa Cruz Biotechnology, whereas anti-pSTAT3 (Y705) and anti-PTEN were procured from BD Pharmingen (BD Biosciences, San Jose, CA, USA). As claimed by the manufacturer and subsequently established by us in an electrophoretic mobility shift assay [2], the anti-STAT3 antibody used in present study specifically detects the active form of STAT3. Dulbecco’s modified Eagle’s medium (DMEM) was obtained from Invitrogen, (Life Technologies CA, USA), fetal calf serum (FCS), MTT [3-(4, 5-dimethylthiazol-2-yl)-2, 5-diphenyltetrazolium bromide], penicillin streptomycin solution, were obtained from Sigma-Aldrich Chemicals (St Louis, MO, USA). All other reagents were of analytical grades and were procured from Sigma-Aldrich unless specified.

### Cell line

Cervical cancer cell line, SiHa (HPV16-positive) was procured from ATCC and was maintained in prescribed culture conditions in DMEM with 10% FCS and 1× penicillin-streptomycin solution. Cells were cultured and treated at sub-confluent density (<30% confluence). Cells were free of mycoplasma as tested by DAPI staining. Moreover, these cells are positive for HR-HPV16 and are known to express full length E6 [23]. Technically, these cells were easier to transfect than other cervical cancer cells available and thus were used throughout present study.

### MTT assay

The viability of treated cells was examined by MTT assay on cells using standard procedure routinely followed in the laboratory [24]. The cells (5000 c/w) were incubated in triplicate in a 96-well plate in the presence or absence of indicated test agent in a final volume of 0.1 ml for desired durations at 37°C. Thereafter, 0.025 ml of MTT solution was added to each well. After 2 h incubation at 37°C, the lysis buffer (20% SDS 50% dimethyl formamide) was added, the plate was incubated overnight at 37°C for solubilization of formazan crystals. The OD at 570 nm was measured using a 96 well multi-scanner autoreader (Biotek, Winooski, USA) with the lysis buffer serving as blank and wells lacking cells was used for background subtraction. The percentage of cell viability was calculated using the following formula:


### Transient transfection

For STAT3 and HPV16 E6 RNA interference assay or transfection of miR-21 inhibitor and Let-7a mimic oligos, SiHa cells (2 × 10^5^ c/w for 6-well in 2 ml or 1000 c/w in 0.1 ml for 96-well plate) were seeded in antibiotics-free normal growth medium supplemented with 10% FCS and incubated at 37°C in a CO_2_ incubator for 18-24 h until the cells were 30% confluent. Next day, the cells were transfected using RNAiMax reagent. For each well to be transfected, RNAi duplex-Lipofectamine RNAiMax complexes were prepared using varying concentrations of siRNA against STAT3, HPV16 E6 (20-80 nM) or miR-21 inhibitor and Let-7a mimic (0.1 nM-50 nM) according to the manufacturer’s instructions. Cells were incubated for 48 h and photographed in culture prior to their end-point assay.

### Isolation of total RNA and amplification of microRNAs by real time-PCR (qRT-PCR)

Upon completion of the indicated time period, cultures were terminated by washing the cells with ice cold PBS, prior to isolation of the RNA to remove dead or dying cells that get detached or get loose. Total RNA from treated and control cells was extracted using mirVana miRNA isolation kit Ambion (Austin, Texas, USA) as per the manufacturer’s instructions. microRNAs expression was checked by using mirVana qRT-PCR kit and miR-21 and Let-7a-specific primers (Ambion) as per the kit protocol. PCR was performed using BioRad iCycler real-time PCR (BioRad, USA). U6 RNA (Ambion) was used for normalization. Data were expressed as absolute fold change with respect to control that was calculated 2^-ΔΔCt^ method. Primer sequences used for miR expression analysis are given in Additional file 1: Table S1.

### Isolation of total proteins from cervical cancer cell lines and immunoblotting

Upon completion of the indicated time period, cultures were terminated by washing the cells with ice cold PBS, prior to isolation of total cellular proteins. Total proteins from cervical cancer cell lines were prepared by the method described previously [2]. Total cellular proteins (50 μg/lane) were separated in 8-12% polyacrylamide gel and electro-transferred on PVDF membrane (Millipore Corp, USA). The membrane was probed with primary antibody of interest. These blots were washed, incubated with HRP-anti-mouse IgG secondary antibodies and visualized by ECL detection kit (Santa Cruz) and by exposing the blot to KODAK X-Omat films (Kodak, India). The membranes were re-probed for β-actin expression as an internal control. The quantitative densitometric analysis of the bands was performed using AlphaEase FC version 4.1.0 (AlphaInnotech Corporation, IL, USA). Normalized fold difference in expression of STAT3/pSTAT3 and other proteins in each sample was determined by densitometric analysis of each band in comparison to band in untreated culture and normalized for input by using respective β-actin bands.

### Reverse transcription-PCR (RT-PCR) for STAT3 mRNA expression

Total RNA was isolated from treated cells using mirVana microRNA isolation kit as described previously and subjected to reverse transcription using Omniscript RT-PCR kit (Qiagen, Hilden, Germany) to prepare the cDNA as per manufacturer instructions [2]. Amplification of STAT3 and GAPDH transcripts from the cDNA was performed as per routine laboratory procedure. Normalized fold difference in expression of STAT3 transcripts in each sample was determined by densitometric analysis of each band in comparison to band in untreated culture and normalized for input by using respective GAPDH bands.

### Statistical analysis

Student’s t-test was employed to analyze the significant difference between control and treated cells. *p* value <0.05 was considered significant. SPSS V16 software was used for all statistical calculations.

## Results

### Targeting STAT3 expression in cervical cancer cells abrogates miR-21 expression

**Figure 1:**
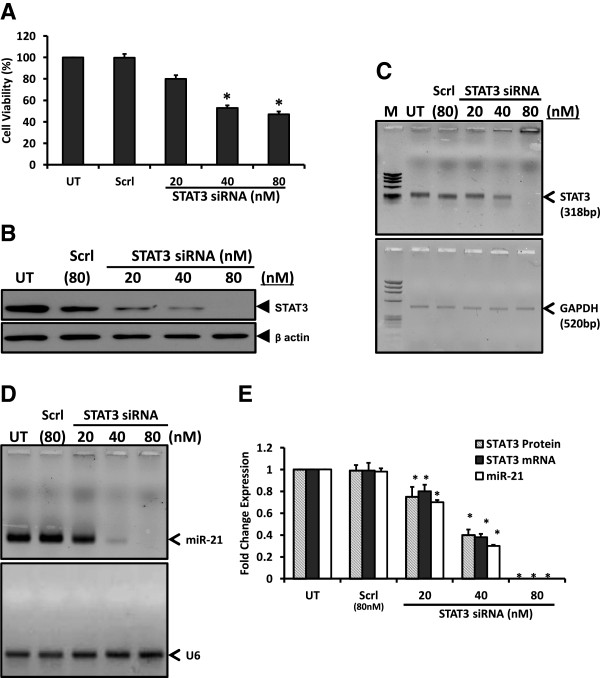
**Effect of targeting STAT3 expression by RNA interference on miR-21 expression.** SiHa cells (2 × 10^5^ cells) transiently-transfected with indicated concentrations of STAT3-specific siRNA for 48 h were examined for viability, STAT3 protein and transcript levels and expression of miR-21. Scrambled siRNA (Scrl) was used as control. **A**. Graph showing SiHa cell viability by MTT assay following transient transfection at indicated doses of STAT3 siRNA. Values are mean ± SD of triplicate cultures with respect to untreated control. *p value <0.05. **B** &**C**. Dose-dependent effect of STAT3-siRNA on STAT3 expression. Cellular proteins (50 μg/lane) isolated from transfected SiHa cells were examined for STAT3 protein expression by immunoblotting **(B)**. Blots were stripped and re-probed with β-actin antibody as loading control. **(C)** Representative Ethidium bromide-stained agarose gel (3%) photograph showing levels of STAT3 transcripts measured by RT-PCR (upper panel) in cDNA derived from STAT3 siRNA-treated SiHa cervical cells. GAPDH RT-PCR was used as internal control for input RNA (lower panel). M: ΦX 174 HaeIII-digested molecular weight markers; UT-untreated cells. **D** &**E**. Inhibition of STAT3 levels accompanied loss of cellular miR-21 levels. Total miRNA pool isolated from treated SiHa cells was examined for levels of miR-21 by qRT-PCR as described in ‘Methods’. Level of ubiquitously-expressed microRNA U6 similarly amplified in parallel was used as input control of miRNA. **(D)**. Specific STAT3 bands were evaluated densitometrically and normalized against untreated control (UT). miR-21 fold change was calculated with respect to control by 2^-ΔΔCt^ method. Graph showing mean ± SD of the fold change in expression of STAT3 protein, STAT3 transcripts and miR-21 after STAT3 inhibition in three independent experiments **(E)**. *p value <0.05 compared to untreated cells.

To test the STAT3-mediated regulation of miR-21, first we performed *in vitro* silencing of STAT3 expression in cervical cancer cells, SiHa, using siRNA against STAT3. SiHa cells were transiently transfected with a pool of STAT3-specific siRNA at 20, 40, and 80 nM concentrations at 48 h. Treated cultures showed altered cell morphology which was accompanied by significant loss of cell viability at 40nM or higher doses (Figure 1A). Moreover, when examined for STAT3 protein level, cells remained in culture were found with decreased level of STAT3 proteins in a dose-dependent manner (Figure 1B). Inhibition of STAT3 expression was observed at concentrations as low as 20 nM and was completely abolished at 80 nM. These effects were STAT3-specific as control siRNA-treated cells did not lose their viability at similar doses of scrambled siRNA. To reconfirm that the STAT3 inhibition is at the transcript level, cDNA prepared from treated cells were further analyzed by reverse transcriptase PCR. As shown in Figure 1C, cells treated with STAT3 siRNA expressed low level of transcripts. Subsequently these cells were subjected to miR-21 expression analysis to study the cellular effects of STAT3 silencing. Interestingly, dose of STAT3 siRNA that abrogated STAT3 expression resulted in a dose-dependent decline of miR-21 expression in treated-SiHa cells, whereas endogenous level of house-keeping gene U6 remained unaltered (Figure 1D). Altogether, decline in cellular STAT3 level were accompanied by reduced expression of miR-21 (Figure 1E).

### Inhibition of phospho-STAT3 Tyr(705) by curcumin and Stattic abrogates miR-21 expression

**Figure 2:**
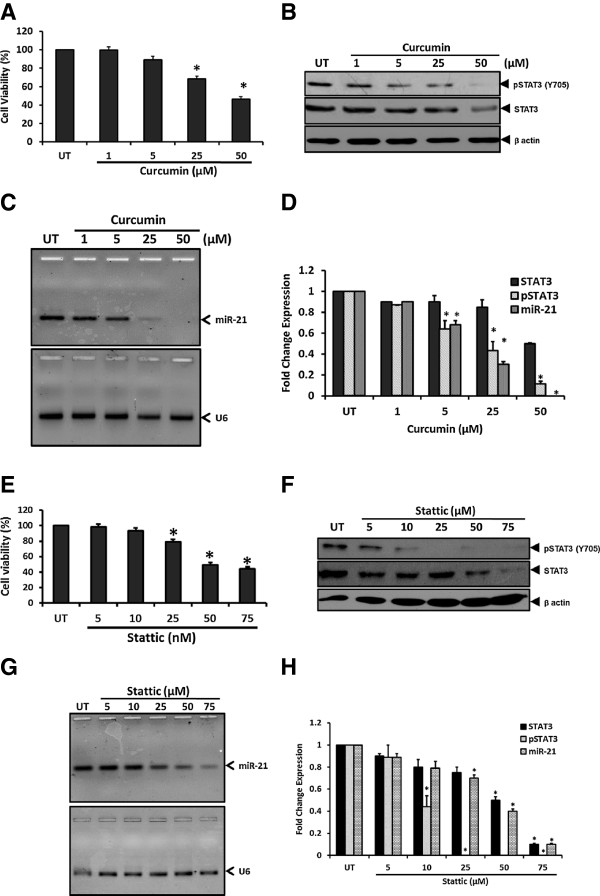
**Effect of pharmacological inhibition of constitutively active STAT3 by curcumin or phospho-STAT3 (Y705)-specific inhibitor stattic on miR-21 expression.** SiHa cells (5 × 10^5^ cells/w) seeded overnight were treated with indicated concentrations of curcumin or stattic for 24 h. Cellular proteins of the treated cells were examined for pSTAT3 (Y705), STAT3 and β-actin by immunoblotting or their RNA was checked for level of miR-21 and U6. Cells were treated in parallel in a 96 well plate to evaluate effect of treatment on cell viability by MTT assay. **A** &**E**. Graph showing cell viability by MTT assay following treatment with indicated doses of curcumin **(A)** or stattic **(E)**. Values are mean ± SD of triplicate cultures with respect to untreated control. *p value <0.05. **B** &**F**. Dose-dependent effect of curcumin **(B)** or stattic **(F)** on levels of pSTAT3 (Y705). Cellular proteins (50 μg/lane) isolated from SiHa cells were examined first for pSTAT3 expression by immunoblotting, following which the blots were stripped and re-probed sequentially with STAT3 and β-actin antibodies as control. **C**, **D**, **G** &**H**. Inhibition of pSTAT3 levels accompanied loss of cellular miR-21 levels. Total miRNA pool isolated from SiHa cells treated with curcumin **(C)** or stattic **(G)** were examined for miR-21 by qRT-PCR. Expression of U6 similarly amplified in parallel was used as input control. Specific pSTAT3 and STAT3 bands were evaluated densitometrically and normalized against untreated control (UT). miR-21 fold change was calculated with respect to control by 2^-ΔΔCt^ method. Graph showing mean ± SD of the fold change in expression of pSTAT3, total STAT3 and miR-21 after inhibition of STAT3 phosphorylation in three independent experiments **(D & H)**. *p value <0.05 compared to untreated cells.

Considering the regulatory role of Tyr(705) phosphorylation in dimerization, nuclear translocation and DNA-binding of STAT3 that initiate downstream signaling, we attempted inhibition of constitutively active STAT3 signaling in cervical cancer cells by blocking STAT3 Tyr(705) phosphorylation using two different inhibitors, curcumin, or Stattic. Among these, curcumin, a strong but non-specific inhibitor of STAT3 phosphotyrosination at Y705 that control STAT3 dimerization, nuclear translocation and subsequent DNA-binding and transactivation; has been shown to manifest its effect through blocking upstream STAT3 signaling [24, 25]. On the contrary, Stattic selectively inhibits the function of the STAT3 SH2 domain regardless of the STAT3 activation state in vitro and selectively inhibits activation, dimerization, and nuclear translocation of STAT3 [26]. SiHa cells treated with increasing concentrations of curcumin or Stattic for 24 h demonstrated reduction in number of cultured cells at 25 μM dose or higher. A proportion of curcumin or Stattic-treated cells showed morphology of a typical apoptotic cell and dose-dependent loss of cell viability was evident from MTT assay (Figure 2A and E). Curcumin and Stattic-treated cells when examined for pSTAT3(Y705) by immunoblotting using specific monoclonal antibody that recognizes only the phosphorylated STAT3 and subsequently with STAT3 antibody that detects total STAT3 pool revealed a dose-dependent decline in cellular level of active pSTAT3 in treated cells. At lower concentrations, the inhibitory effect was observed specifically on pSTAT3 level whereas the total STAT3 remained unaltered indicating specific effect of the inhibitors on STAT3 phosphorylation at low doses only. However, at higher concentrations of inhibitors, a simultaneous decline in overall STAT3 pool was observed (Figure 2B and F). Treatment of cells at 25 μM Stattic resulted in complete loss of phosphorylation at the tyrosine residue 705 (Figure 2F). Further, the cells treated with curcumin or Stattic were analyzed for miR-21 expression by quantitative real time PCR analysis. Curcumin or Stattic at doses that specifically blocked STAT3 phosphorylation (upto 25 μM) abrogated miR-21 level (Figure 2C, D and G, H).

### Silencing of miR-21 promotes accumulation of PTEN and abrogates pSTAT3 level

**Figure 3:**
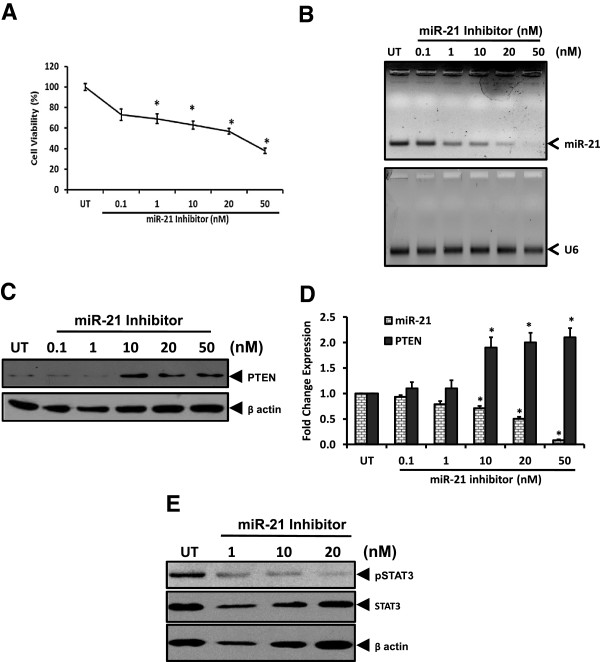
**Effect of miR-21 silencing on cell viability, on expression of PTEN and active STAT3 level.** SiHa cells transiently-transfected with indicated concentrations of miR-21 – specific inhibitor for 48 h were examined for viability and expression of downstream target PTEN. **A**. Graph showing cell viability by MTT assay following transient transfection with miR-21 inhibitor at indicated doses. Values are mean ± SD of triplicate cultures with respect to untreated control. *p value <0.05. **B**. Representative gel photograph showing reduced levels of miR-21 in treated cells (upper panel). Total miRNA pool isolated from treated SiHa cells was examined for miR-21 by qRT-PCR. Expression of U6 similarly amplified in parallel was used as input control (lower panel). M: ΦX 174 HaeIII-digested molecular weight markers; UT-untreated cells. **C** &**D**. Dose-dependent accumulation of PTEN in cells treated with miR-21 inhibitor. Cellular proteins isolated from treated SiHa cells were examined first for PTEN expression by immunoblotting, following which the blots were stripped and re-probed with β-actin antibody as loading control **(C)**. Specific PTEN bands were evaluated densitometrically and normalized against untreated control (UT). miR-21 fold change was calculated with respect to control by 2^-ΔΔCt^ method. Graph showing mean ± SD of the fold change in expression of miR-21 and PTEN protein after inhibition of miR-21 in three independent experiments **(D)**. *p value <0.05 compared to untreated cells. **E**. Concomitant reduction of pSTAT3 by miR-21 inhibitor. Cellular proteins isolated from treated SiHa cells were examined first for pSTAT3 and STAT3 expression by immunoblotting, following which the blots were stripped and re-probed with β-actin antibody as loading control.

To assess functional contribution of miR-21 in constitutive activation of STAT3 in cervical cells, we experimentally knocked down the cellular miR-21 using miRIDIAN microRNA inhibitor specific for miR-21 and checked its effects on its downstream target and negative regulator of STAT3 phosphorylation, PTEN. Cells transfected with miR-21 inhibitor revealed growth inhibition till 20 nM. However, beyond this concentration this inhibitor was cytotoxic and cells started showing cellular alterations resembling apoptosis with decline in overall cell numbers which was confirmed in MTT assay (Figure 3A). qRT-PCR analysis of total RNA isolated from treated cells showed loss of cellular miR-21 level with increasing dose of the inhibitor. At high doses of inhibitor (50 nM), expression of control miRNA U6 was partially affected (Figure 3B). Immunoblot analysis of these cells for PTEN expression revealed a dose-dependent increase in cellular PTEN pools in miR-21 inhibitor-treated cells. Treated cells showed significant increase in PTEN level at 10 nM dose but a further increase in inhibitor dose did not enhance PTEN level (Figure 3C). Treated cells showed significant increase in PTEN level at 10nM dose but a further increase in inhibitor dose did not enhance PTEN level (Figure 3D). Further analysis of miR-21 inhibitor treated cells for levels of pSTAT3 and STAT3 revealed a dose-dependent decrease in STAT3 activation whereas STAT3 expression was only marginally affected (Figure 3E).

### Let-7a negatively regulates STAT3 expression in cervical cancer cells

**Figure 4:**
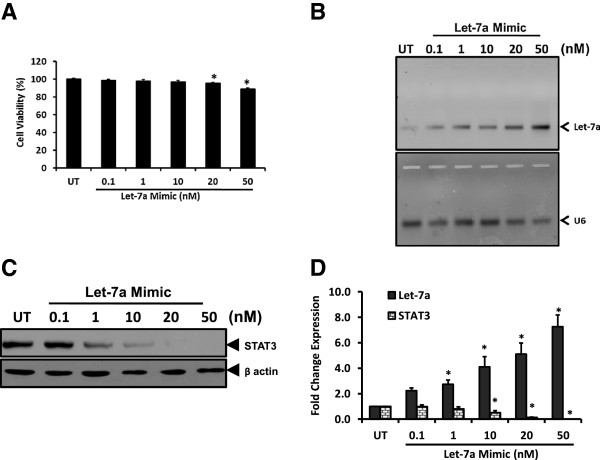
**Effect of transiently expressed Let-7a mimic on cellular STAT3 pools.** SiHa cells (2 × 10^5^ cells) transiently-transfected with indicated concentrations of Let-7a mimic for 48 h were examined for viability, intracellular Let-7a and STAT3 expression. **A**. Graph showing cell viability by MTT assay following transient transfection with Let-7a mimic at indicated doses. Values are mean ± SD of triplicate cultures with respect to untreated control. *p value <0.05. **B**. Representative gel photograph showing increased intracellular levels of Let-7a in treated cells (upper panel). Total RNA isolated from treated SiHa cells was examined for Let-7a by qRT-PCR. Expression of U6 similarly amplified in parallel was used as input control (lower panel). M: ΦX 174 HaeIII-digested molecular weight markers; UT-untreated cells. **C** &**D**. Dose-dependent effect of transiently-transfected Let-7a on STAT3 levels. Cellular proteins isolated from Let-7a mimic-treated SiHa cells were examined for levels of STAT3 protein by immunoblotting **(C)**. Blots were stripped and re-probed with β-actin antibody as loading control. Specific STAT3 bands were evaluated densitometrically and normalized against untreated control (UT). Let-7a fold change was calculated with respect to control by 2^-ΔΔCt^ method. Graph showing mean ± SD of the fold change in expression of intracellular Let-7a and STAT3 protein after treatment with Let-7a mimic in three independent experiments **(D)**. *p value <0.05 compared to untreated cells.

In the next part of our study, we assessed whether Let-7a has any regulatory role in controlling STAT3 level in cervical cells. As basal Let-7a level in SiHa cells were very low, we augmented the level by intracellular delivery of chemically-enhanced Let-7a mimic that functions as endogenous mature miRNA, by transient transfection for 48 h. Cells treated with Let-7a mimic did not show any peculiar change in morphology of the cultured cells. However, growth of these cells appeared marginally inhibited which was evident only at higher concentrations of Let-7a mimic (Figure 4A). Measurement of Let-7a in treated cells by qRT-PCR revealed a dose-dependent increase in intracellular Let-7a pools in mimic-treated cells (Figure 4B). Immunoblotting of total proteins from these treated cells for assessment of STAT3 proteins revealed a dose-dependent decrease in level of total STAT3 (Figure 4C). Let-7a at doses above 20nM completely abolished cellular STAT3 level in cervical cells. These results demonstrated an inverse correlation between level of Let-7a and STAT3 (Figure 4D).

### Blocking HPV16 E6 oncoprotein results in increased Let-7a, loss of miR-21 expression, loss of active STAT3 with an increase in PTEN level

**Figure 5:**
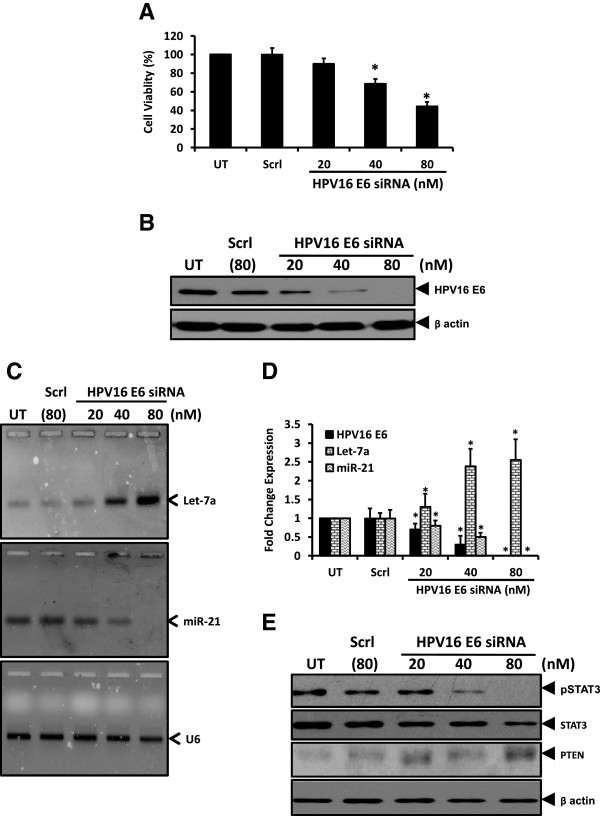
**Effect of targeting HPV16 E6 oncogene on miRs, Let-7a and miR-21 and level of active STAT3.** SiHa cells transiently-transfected with indicated concentrations of HPV16 E6 -specific siRNA for 48 h were examined for viability, expression of Let-7a and miR-21. Scrambled siRNA (Scrl) was used as control. **A**. Graph showing cell viability by MTT assay following transient transfection with E6 siRNA at indicated doses. Values are mean ± SD of triplicate cultures with respect to untreated control. *p value <0.05. **B**. Dose-dependent effect of E6-siRNA on endogenous HPV16 E6 levels. Cellular proteins isolated from transfected SiHa cells were examined for HPV16 E6 protein expression by immunoblotting. Blots were stripped and re-probed with β-actin antibody as loading control. **C** &**D**. Targeting of E6 oncoprotein results in elevation of Let-7a levels and corresponding decline in miR-21.Total miRNA pools isolated from SiHa cells treated with E6-siRNA were examined for Let-7a (upper panel) and miR-21 (middle panel) by qRT-PCR **(C)**. Expression of U6 similarly amplified in parallel was used as input control (lower panel). M: ΦX 174 HaeIII-digested molecular weight markers; UT-untreated cells. Specific HPV16 E6 bands were evaluated densitometrically and normalized against untreated control (UT). Let-7a and miR-21 fold change were calculated with respect to control by 2^-ΔΔCt^ method. Graph showing mean ± SD of the fold change in expression of HPV16 E6, Let-7a and miR-21 after transient transfection of E6 in three independent experiments **(D)**. *p value <0.05 compared to untreated cells. **E**. Effect of targeting HPV E6-siRNA on pSTAT3, STAT3 and PTEN expression levels. Cellular proteins (50 μg/lane) isolated from transfected SiHa cells were examined for expression of indicated proteins by immunoblotting. Blots were stripped and re-probed with β-actin antibody as loading control.

As viral oncogene E6 of high risk HPVs play a vital role in tumorigenic transformation of cervical cells, we assessed the effect of HPV16 E6 silencing on expression of Let-7a, STAT3 and miR-21. SiHa cells transiently transfected with commercially available HPV16 E6 siRNA revealed an E6 transfection-specific growth inhibition and loss of cell viability in treated cultures (Figure 5A). Immunoblotting of proteins from treated cells revealed an effective silencing of the viral oncoprotein E6 (Figure 5B). Analysis of cellular miRNA derived from these treated cells by Let-7a and miR-21 qRT-PCR revealed an increase in cellular Let-7a pool and a corresponding dose-dependent decline in miR-21 level (Figure 5C and D). In contrast, control cells treated with scrambled siRNA failed to show any change in level of Let-7a or miR-21 expression. Thus, HPV16 E6 targeting resulted in a specific up-regulation of Let-7a and abrogation of miR-21 level (Figure 5D). E6-transfected cells were further examined for STAT3 and PTEN levels to establish the existence of a miR-mediated loop. E6-transfected cells showed strong decline in level of pSTAT3 in dose-dependent manner with complete loss of pSTAT3 at 80nM of E6 siRNA (Figure 5E). In addition to loss of tyr phosphorylation there was a gradual decline in the overall level of STAT3 protein. Loss of pSTAT3 was accompanied by gain of PTEN level in E6-siRNA treated cervical cancer cells.

## Discussion

In the present study, we examined the role of specific miRs, miR-21 and Let-7a in aberrantly expressed STAT3 signaling during cervical carcinogenesis. STAT3 was targeted by different experimental approaches i.e. silencing the expression using specific siRNA, or abrogation of its Tyr(705) phosphorylation using a broad spectrum inhibitor curcumin or a specific rationally-designed small molecule inhibitor, Stattic. Irrespective of the experimental strategy, knocking down STAT3 resulted in a dose-dependent decline in level of miR-21. Indirect inhibition of miR-21 by targeting STAT3 or its direct inhibition using specific hairpin inhibitor reduced viability of cervical cancer cells and resulted in a dose-dependent accumulation of PTEN. On the contrary, when Let-7a pool of the cells was augmented by intracellular delivery of a stable chemically-synthesized Let-7a oligonucleotide mimic, which structurally and functionally resembled endogenous Let-7a, the treated cells showed a sharp decline in cellular level of STAT3 protein which was Let-7a dose-dependent. Further, blocking of oncogenic E6 gene expression in cervical cancer cells by E6-specific siRNA resulted in an increase in level of Let-7a and corresponding decline in miR-21 which was accompanied by loss of active STAT3 and increase in PTEN in E6-transfected SiHa cells.

### Blocking of STAT3 expression by siRNA abrogates miR-21 expression

Inhibitory effect of silencing STAT3 signaling on miR-21 and cell viability demonstrates a potential role of active STAT3 and over-expressed miR-21 in survival and growth of cervical cancer cells. Loss of any of these mediators was found detrimental and the observations made in the present study indicate existence of a positive regulatory loop. Elevated level of miR-21 has been reported in cervical cancer and its role as an oncomiR has been established previously [11, 27, 28]. However, the reason for elevated miR-21 in cervical carcinogenesis remained poorly understood. Apparently, miR-21 forms a critical component of STAT3-mediated regulatory circuits that link inflammation to cancer [29]. Though STAT3-mediated transcriptional regulation of miR-21 is possibly through binding to the promoter [14], the mechanism(s) how miR-21 influences STAT3 signaling remained unexplored. Interestingly, recent study in an analogous system, enforced expression of miR-21 triggered STAT3 signaling [30]. Leads obtained from our miR-21 inhibition experiments demonstrated that miR-21 is involved in a positive feedback loop through down-regulation of PTEN that keeps STAT3 activity under control. PTEN is a negative regulator of STAT3 activation in HPV-infected cells and possesses pTyr(705) STAT3 phosphatase activity which is independent of the kinase activity [16]. miR-21 is known to regulate PTEN expression in hepatocellular carcinoma [15] and overexpression of miR-21 results in targeting of cellular PTEN pools in endometrial cancers [31]. It is likely that STAT3-induced miR-21 contributes to an important part of positive feedback loop in cervical cancer cells that keeps various apoptosis-inducing death regulators including PTEN, under control and miR-21 inhibition alleviates PTEN suppression leading to abrogated STAT3 signaling. Interestingly, STAT3 activation of miR-21 via PTEN has been described as a part of an epigenetic switch that links inflammation to cancer [29]. It is expected that in the absence of dephosphorylation the pSTAT3 remains active longer and maintain its intracellular pools at high level. These observations suggest critical role of miR-21 in down regulation of PTEN and maintenance of constitutively active STAT3 signaling.

### Let-7a acts as negative regulator of STAT3

Following the intracellular delivery of Let-7a mimic, we observed silencing of STAT3 expression. However, the silencing in this case was not manifested by significant loss of cell viability. The reasons behind such discrepant results are difficult to explain. Let-7a has contrasting actions on STAT3 which may be attributed to pleiotropy and redundancy in action of this miRNA. On the one side, it down-regulates the expression of STAT3 at mRNA level [17] and directly inhibits IL-6 expression [32]. On the other hand, there are reports that indicate involvement of Let-7a with constitutively active and phosphorylated STAT3 through NF-2 (neurofibromatosis-2) [33]. Nevertheless, downregulation of Let-7a is observed in cervical cancer lesions [28]. We observed that STAT3 expression and specifically its activation were decreased in response to Let-7a restoration in cervical cancer which is indicative of a negative regulation of STAT3 by Let-7a in cervical cancer cells. Consistent with our observation, restoration of Let-7 expression has been found associated with reduction in tumor growth in those cancers in which the Let-7 family is globally decreased [34, 35], and a reduced expression of Let-7a was found associated with poor prognosis and shortened post-operative survival [19], thus suggestive of a tumor suppressive role of Let-7a during cervical carcinogenesis that may be manifested through control of aberrant STAT3 signaling.

### HPV16 oncoprotein E6 controls STAT3 signaling through a regulatory circuit mediated by Let-7a, miR-21 and PTEN

Recent studies have illustrated a possible interaction between HPV oncoprotein E6 and cellular microRNAs particularly miR-23b, miR-34a and miR-218 [36–38]. However, its effects on miR-21 and Let-7a remained elusive. Our results demonstrate that an E6-specific silencing not only down-regulates miR-21 but also leads to accumulation of Let-7a. These alterations were associated with loss of active STAT3, increase in PTEN level and reduced viability of cervical cancer cells. Silencing of E6/E7 has been reported to induce growth inhibition, loss of transformed phenotype, induces apoptosis and replicative senescence in cervical cancer cells and inhibited tumor formation in animal models [39, 40]. Since knockdown of E6 expression in cervical cells resulted in loss of STAT3 regulatory circuitry, it is likely that constitutively active STAT3 critically requires expression of viral oncoprotein to maintain its associated miR profiles. During the course of infection, some of the viral derivatives are known to alter cellular miRNA profiles. HIV-1 was reported to globally suppress host miRNA expression [41]. The Epstein-Barr virus latent membrane protein 1 was reported to activate miRNA-155 transcription [42]. Similarly, hepatitis B viral protein, HBx, also plays a role in deregulating cellular miRNAs in hepatocellular carcinoma [17]. Why HPV oncoproteins or other pathogenic viral infections activate and sustain STAT3 signaling is still unknown. It is likely that active STAT3 is essential for expression of viral oncogenes. A strong correlation of active STAT3 with levels of E6/E7 in cervical cancer lesions, and specific silencing of STAT3 resulting in abrogated E6 expression in cervical cancer have been observed earlier [1, 3]. Taken together, these leads are suggestive of a cooperative interaction between HPVE6 and STAT3 signaling that may have a carcinogenic outcome.

Interestingly, loss of E6 also translated in accumulation of Let-7a. In view of the fact that Let-7a is a negative regulator of STAT3 transcript, loss of E6 and resultant build-up of Let-7a pool result in a stronger silencing of STAT3 which is also manifested at the transcript level. Although there is no report that implicate E6 in reducing cellular Let-7a, a decline of Let-7a is frequently reported in cervical neoplastic tissues [28, 43]. Further studies are needed to understand the inhibitory effect of E6 on Let-7a. In view of the fact that E6 is known to activate IL-6/STAT3 signaling in cervical cancer cells [22], our results demonstrate existence of HPV16 E6-induced proinflammatory signaling that operate through STAT3 and miR-21 by blocking its negative regulators Let-7a and PTEN.

## Conclusions

Overall, we demonstrate for the first time, an existence of miRNA-mediated loop involving miR-21 and Let-7a, which are positively fed by viral oncoprotein E6 and responsible for aberrant STAT3 signaling during HPV-induced cervical carcinogenesis. Therefore, silencing STAT3 by siRNA, curcumin and Stattic, and targeting miR-21 by antisense or small-molecule compounds may represent a potential therapeutic strategy for targeted treatment of cervical and other cancers which invariably overexpress STAT3.

## Electronic supplementary material

Additional file 1: **List of primers used for microRNA-21 & Let-7a qRT PCR and STAT3 RT-PCR in the study.** (DOCX 20 KB)

## Abbreviations

HPV: Human papillomavirus
miR: microRNA
STAT3: Signal transducer and activator of transcription
PTEN: Phosphatase and tensin homologue
siRNA: Small interfering RNAs.
