# Supplementary material for: Deregulation of microRNAs Let-7a and miR-21 mediate aberrant STAT3 signaling during human papillomavirus-induced cervical carcinogenesis: role of E6 oncoprotein
Source: BMC Cancer. 2014 Dec 23;14:996. doi: 10.1186/1471-2407-14-996 (PMC4364636; doi:10.1186/1471-2407-14-996)
Supplement: Supplementary file 1 — Additional file 1: List of primers used for microRNA-21 & Let-7a qRT PCR and STAT3 RT-PCR in the study. (DOCX 20 KB) [file 12885_2014_5181_MOESM1_ESM.docx]

**Supplementary Table I- List of primers used for microRNA-21 & Let-7a qRT PCR and STAT3 RT-PCR in the study**

| **Primer Name** | **Amplicon Size (bp)** | **Primer Sequence** |
| --- | --- | --- |
| **U6 RT** | **90** | 5' CGCGCCTGCAGGTCGACAATTAACCCTCACTAAAGGGttgcgtgtcatcc 3' |
| **U6 PCR(F)** |  | 5' GTAATACGACTCACTATAGGGAGAAGAGcctgcgcaagg 3' |
| **U6 PCR(R)** |  | 5' CGCGCCTGCAGGTCGAC 3' |
| **miR-21 RT** | **90** | 5' CGCGCCTGCAGGTCGACAATTAACCCTCACTAAAGGGtcaacatcagtct 3' |
| **miR-21 PCR(F)** |  | 5' GTAATACGACTCACTATAGGGAGAAGAGtagcttatcag 3' |
| **miR-21 PCR(R)** |  | 5' CGCGCCTGCAGGTCGAC 3' |
| **Let-7a RT** | **90** | 5' CGCGCCTGCAGGTCGACAATTAACCCTCACTAAAGGGaactatacaacct 3' |
| **Let-7a PCR(F)** |  | 5' GTAATACGACTCACTATAGGGAGAAGAGtgaggtagtag 3' |
| **Let-7a PCR(R)** |  | 5' CGCGCCTGCAGGTCGAC 3' |
| **STAT3 RT(F)** | **318** | 5’-TTGCCAGTTGTGGTGATC-3’ |
| **STAT3 RT(R)** |  | 5’-GAACCCAGAAGGAGAAGC-3’ |
| **GAPDH RT(F)** | **322** | 5’-TGGATATTGTTGCCATCA ATGACC-3’ |
| **GAPDH RT(R)** |  | 5’-GATGGCATGGACTGTGGTCATG-3’ |

The capital letters indicate universal primers sequence of miRNAs for RT and forward primer of each miR while small letters sequence is specific for the miRNA of interest which binds to the miRNA. The amplicon size for each miR is 90bp.

|  |  |  |
| --- | --- | --- |
|  |  |  |
